# Studying Several Properties of Emulgel Based on κ‐Carrageenan and Its Incorporation as Shortening Material in Cookies

**DOI:** 10.1002/fsn3.71210

**Published:** 2025-11-18

**Authors:** Mahdieh Sharifi, Niloofar Moshfegh, Azam Abbasi

**Affiliations:** ^1^ Nutrition Research Center, Department of Food Hygiene and Quality Control, School of Nutrition and Food Sciences Shiraz University of Medical Sciences Shiraz Iran

**Keywords:** bakery, fat substitute, gel‐based systems, hydrocolloid, rheological properties

## Abstract

Using liquid oils rich in unsaturated fats is a healthier alternative to saturated fats like shortening; however, their high unsaturation can impair texture and oxidative stability in baked goods. Structuring these oils into gel‐like emulgels helps overcome these limitations. In this study, κ‐carrageenan emulgels containing 30% sunflower oil and different κ‐carrageenan levels (0.50%, 1.00%, and 1.50%) were formulated and evaluated for their textural, rheological, and other properties. The 1.50% κ‐carrageenan emulgel showed higher hardness (3.25 N) but lower springiness (2.76 mm) than the 1.00% emulgel (1.44 N, 3.14 mm), indicating an inverse hardness–springiness relationship. Dynamic rheology showed that the 1.50% emulgel had an earlier crossover at 14.6% strain, indicating higher brittleness than the 1.00% emulgel (61.8%). The 0.50% emulgel lacked sufficient strength, while the 1.50% was too firm for cookie batter. Therefore, the 1.00% formulation was selected for testing as a 50% and 100% shortening replacement in cookies. Cookies were prepared and compared with controls made with 100% shortening or liquid oil, evaluating properties such as texture and peroxide levels. Cookies with 50% and 100% emulgel substitution showed 30% and 50% reductions in fat content, respectively, compared to controls. During one month of storage, cookies with 100% emulgel exhibited a substantial increase in hardness (from 12.88 N to 30.70 N), while those with 50% emulgel maintained stable hardness. Cookies containing emulgel exhibited greater oxidative stability than the control throughout storage. Overall, the 50% emulgel replacement yielded the most favorable results, highlighting its potential as a shortening substitute in cookies.

## Graphical abstract

The emulgel contained 1% κ‐carrageenan was selected as optimum emulgel system for shortening substitution in cookie formulation. Produced cookie by 50% shortening and 50 emulgel was similar to control cookie containing 100% shortening from point of physicochemical properties.

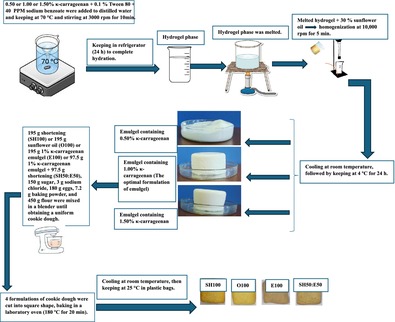

## 1 Introduction

Shortening is one of the main ingredients in cookie formulations, providing lubrication, flavor, and texture (Maache‐Rezzoug et al. 1998). Traditionally, bakery shortenings are rich in saturated fats (SF), such as palmitic, stearic, and oleic acids (Rangrej et al. 2015). A major concern with baked cereal products, therefore, is their high solid fat content (Sanz et al. 2017). Dietary saturated fats are associated with adverse health effects, including an increased risk of coronary heart disease (CHD) and cardiovascular disease (CVD) (De Souza et al. 2015). Current dietary guidelines recommend reducing SF intake to no more than 10%–11% of total daily energy and replacing them with unsaturated fats (UF) (Paciulli et al. 2020).

Liquid vegetable oils, which are high in unsaturated fats, are a healthier alternative to saturated fats in baked products. However, their high degree of unsaturation and low viscosity can negatively affect aeration, texture, and oxidative stability in baked goods (Manaf et al. 2019; Pehlivanoğlu et al. 2018). One strategy to overcome these limitations is to convert liquid oils into gel‐like structures, which can protect the oil against oxidation and other chemical deterioration, thereby improving the shelf life and quality of baked goods (Martín‐Illana et al. 2022; Pehlivanoğlu et al. 2018).

Emulgels—gelled oil‐in‐water emulsions—are particularly promising in this context. They resemble solid fats in terms of mechanical and textural properties, while offering the added advantage of requiring significantly less oil than other gel‐based structures such as oleogels (Lu et al. 2019; Zare et al. 2023). In recent years, emulgels have been widely applied as fat replacers in different areas of the food industry. For instance, Pintado et al. (2021) incorporated emulgels containing polyphenol extracts into frankfurter sausages, while Mefleh et al. (2022) developed spreadable cheese using pea protein–olive oil emulsion‐filled gels. Similarly, Li, Zhang, et al. (2022) produced yoghurts with emulgels based on whey proteins and vegetable oils as fat replacers.

In bakery products, several studies have demonstrated that cookies containing emulgels exhibit reduced fat content and improved oxidative stability compared to those made with shortening or butter. For example, Gutiérrez‐Luna et al. (2023) substituted butter with 100% olive oil–alginate gelled emulsion in cookies and reported a 40% reduction in fat content compared with butter cookies. After 21 days of storage, the reformulated cookies showed no oxidation issues (< 1.5 nmol/g). Likewise, Giarnetti et al. (2015) used an emulsion‐filled gel based on inulin and extra virgin olive oil as a fat replacer in cookies. They observed a 19% and 46% reduction in total fat when butter was replaced at 50% and 100%, respectively, compared with controls. They concluded that gel‐based systems could serve as suitable alternatives to high saturated fat in cookie formulations.

Sunflower oil contains nearly 80% unsaturated fatty acids and is particularly rich in essential fatty acids, especially oleic acid, which accounts for 85%–95% of its total fatty acid content (Qammar‐uz‐Zaman et al. 2023). Carrageenan is a high‐molecular‐weight sulfated polysaccharide extracted from red seaweed. Among its types, κ‐carrageenan can form reversible gels through the association of two or three double helices during gelation (Yuguchi et al. 2002). Gel network formation by hydrocolloids such as κ‐carrageenan enhances both viscosity and emulsion stability (Lee et al. 2023).

κ‐carrageenan‐based emulgels have already been used to replace animal fats in various food products—for instance, in sausages (Fontes‐Candia et al. 2023; Li, Meng, et al. 2022) and cheeses (Ghiasi and Golmakani 2022; Rojas‐Nery et al. 2015). Nutter et al. (2023b) replaced commercial fats (butter and shortening) in shortbread with bigels composed of a rice bran wax–soybean oil oleogel and an alginate/κ‐carrageenan hydrogel. Their reformulated shortbreads exhibited comparable moisture content and water activity, along with greater tenderness, compared to those made with commercial fats.

To the best of our knowledge, however, κ‐carrageenan‐based emulgels have not yet been investigated as fat replacers in cookies. Therefore, the present study had two main objectives: (i) to produce κ‐carrageenan–sunflower oil emulgels with varying κ‐carrageenan concentrations (0.50%, 1.00%, and 1.50% in the emulsion phase) and to evaluate their mechanical, viscosity, and rheological properties; and (ii) to select the optimal formulation and apply it for partial and total substitution of shortening or sunflower oil in cookie dough. The resulting cookies were then analyzed for fat content, color, and lipid oxidation, and compared with control samples.

## 2 Materials and Methods

### 2.1 Materials

Refined sunflower oil was purchased from Ghoncheh Company (Iran). κ‐carrageenan (100% purity, sulfate group content 15%–40%) was kindly donated by Behin Azma (Iran). Shortening consisted of a blend of sunflower, canola, and soybean oils, obtained from Ladan Co. (Tehran, Iran). Eggs, sodium bicarbonate, baking powder, wheat flour, and sodium chloride were purchased from a local market in Shiraz, Iran. All other chemicals were of analytical grade and obtained from Merck (Darmstadt, Germany).

### 2.2 Emulgels Preparation

Emulgels were prepared following the method of Zare et al. (2023) with minor modifications. To prepare the hydrogel phase, different concentrations of κ‐carrageenan powder (0.50, 1.00, and 1.50 wt%) were separately dissolved in distilled water to form firm gels. Tween 80 (0.1 wt%, as emulsifier) and sodium benzoate (400 ppm, as preservative) were added to the solutions, which were continuously stirred at 3000 rpm while maintaining a temperature of 70°C for 10 min. The solutions were then refrigerated at 4°C for 24 h to ensure complete hydration. For emulgel preparation, the hydrogels were remelted using a heater, and sunflower oil (30 wt%) was incorporated while homogenizing at 20,000 rpm for 5 min using a high‐speed homogenizer (Heidolph, Germany). The emulgels were cooled to room temperature and subsequently stored at 4°C for 24 h to complete gelation. The final products were stored at 4°C until analysis.

### 2.3 Characteristics of Emulgels

#### 2.3.1 Solvent Holding Capacity (SHC)

The solvent holding capacity of the emulgels was measured according to Martins et al. (2023) with slight modifications. This method quantitatively determines the amount of solvent retained in the emulgel matrix. Freshly prepared emulgel samples (1 mL) were placed in Eppendorf tubes and stored at 4°C for 24 h. The tubes were then centrifuged at 8600 g for 15 min at room temperature (Sigma, Germany). After centrifugation, the tubes were inverted, and the released solvent was collected on filter paper. SHC was calculated using the following equation (1):

$$\text{SHC}\;\left(\%\right)=\left(1-\left(\left(W_{b}-W_{e}\right)-\left(W_{a}-W_{e}\right)/W_{b}-W_{e}\right)\right)\times 100$$

where *W*_*a*_ = weight of the tube with the sample after removing the released solvent, *W*_*b*_ = weight of the tube with the sample before centrifugation, and, *W*_*e*_ = weight of the tube without the sample.

#### 2.3.2 FTIR Spectroscopy

The FTIR spectra of the emulgels and the hydrogel phase (containing 1% κ‐carrageenan) were obtained using an ATR‐FTIR spectrophotometer (Tensor II, Bruker), following the method of Nutter et al. (2023a). Approximately 1 g of each sample was placed on the crystal surface, and spectra were recorded in the range of 4000–400 cm^−1^, with 32 scans accumulated at a resolution of 4 cm^−1^.

#### 2.3.3 Mechanical Properties

The mechanical properties of the emulgels were evaluated following Martins et al. (2023) with minor modifications. A texture analyzer (TA‐XT2, Stable Microsystems, Surrey, UK) equipped with a 4500 g load cell and a trigger load of 3 g was used at room temperature. A double‐compression test was performed, compressing the emulgels to 10% of their original height. The pre‐test, test, and return speeds were all set at 1 mm/s.

#### 2.3.4 Viscosity

The apparent viscosity of the emulgels was measured at room temperature using an MCR 302 rotational rheometer (Anton Paar, Graz, Austria) following the method of Ghiasi and Golmakani (2022). Measurements were carried out over a shear rate range of 0.1–200 s^−1^.

#### 2.3.5 Dynamic Rheological Measurements

Dynamic rheological properties of the emulgels were analyzed with an MCR 302 controlled stress/strain rheometer (Anton Paar, Graz, Austria), following the method of Ghiasi and Golmakani (2022). First, amplitude sweep tests were conducted over a shear strain range of 0.001%–100% at a frequency of 1 Hz to determine the linear viscoelastic region (LVR). Temperature ramp studies were then performed by heating samples from 25°C to 90°C at 5°C/min, followed by cooling to 25°C at the same rate, also at 1 Hz frequency. The storage modulus (G′) and loss modulus (G″) were calculated using RheoCompass software.

#### 2.3.6 Microstructure and Visual Observation

The microstructure of the emulgels was observed using an optical microscope (20× magnification) equipped with a digital camera featuring a 64 MP sensor and f/1.79 lens (Zare et al. 2023). Additionally, macroscopic images of the emulgels were captured with a high‐resolution mobile camera for visual comparison.

### 2.4 Preparation of Cookies

Cookies were prepared using a modified version of the method described by Gharaie et al. (2019). Briefly, 195 g shortening, 150 g sugar, and 3 g sodium chloride were mixed in a blender. Then, 180 g eggs and 7.2 g baking powder were added and blended, followed by the addition of 450 g wheat flour to obtain a uniform dough. The optimal emulgel formulation (containing 1% κ‐carrageenan in the emulsion phase) was used to replace 50% and 100% of the shortening in the cookie dough. Control doughs were prepared using either 100% shortening or 100% sunflower oil. The complete formulations are presented in Table 1. The dough was cut into square shapes (5 × 5 × 0.3 cm) and baked at 180°C for 20 min. After baking, cookies were cooled at room temperature for 30 min, wrapped in plastic bags, and stored at 25°C until further analysis.

**TABLE 1:** Formulation of cookies.

Samples	Ingredients					
	Fat	Sugar	Sodium chloride	Eggs	Baking powder	Flour
E100	195 g emulgel	150 g	3 g	180 g	7.2 g	450 g
SH50: E50	97.5 g emulgel +97.5 g shortening	150 g	3 g	180 g	7.2 g	450 g
SH100	195 g shortening	150 g	3 g	180 g	7.2 g	450 g
O100	195 g sunflower oil	150 g	3 g	180 g	7.2 g	450 g

*Note:* Samples: E100: Cookie dough with 100% fat content of emulgel, SH50: E50: Cookie dough with 50% fat content of emulgel and 50% shortening, SH100: Cookie dough with 100% fat content of shortening (control), and O100: Cookie dough with 100% fat content of sunflower oil (control).

### 2.5 Characterization of the Cookies

#### 2.5.1 Fat Content Analysis

The fat content of the cookies was determined using the Soxhlet extraction method, as described by Giuffrè et al. (2022).

#### 2.5.2 Hardness Evaluation of Cookies

The breaking force of the cookies was measured by a three‐point bending test using a texture analyzer (TA‐XT2, Stable Microsystems, Surrey, UK), following the method of Šarić et al. (2019). Each cookie was supported by a fixture and fractured by a descending probe. The analyzer settings were: pre‐test speed of 1 mm/s, test speed of 0.5 mm/s, return speed of 0.5 mm/s, trigger force of 5 g, and a target distance of 5 mm. The force corresponding to the first peak was taken as the hardness of the cookies. Measurements were conducted on days 1, 10, 20, and 30 after baking, with at least three replicates for each cookie formulation.

#### 2.5.3 Moisture Analysis

Moisture content was determined by drying 5 g of ground cookie samples at 105°C in a laboratory oven until a constant weight was reached, following Boukid et al. (2021). Measurements were performed on days 1, 8, 15, 22, and 29 after baking.

#### 2.5.4 Color Analysis

Cookie color (L*, a*, b* values) was measured by digital photography, as described by Ghiasi et al. (2020). Samples were placed in a sealed wooden box and imaged using a high‐resolution mobile camera.

#### 2.5.5 Cooking Loss (%)

Cooking loss (%) was calculated by comparing cookie weights before and after baking, according to the following equation (2):

(2) $$\begin{array}{c} \text{Cooking loss}\left(\%\right)\\ =\frac{\text{cookie dough weight}\;\left(\text{before baking}\right)-\text{cookie weight}\;\left(\text{after baking}\right)}{\text{cookie dough weight}\;\left(\text{before baking}\right)}\\ \times 100 \end{array}$$

#### 2.5.6 Lipid Oxidation

Oxidative stability of the cookies was evaluated by measuring the peroxide value at 20‐day intervals over a storage period of 110 days, following the method of Kaur et al. (2023) with slight modifications. Briefly, 5 g of sample was mixed with 50 mL chloroform and shaken for 3 h to extract the fat. The extract was then filtered through Whatman No. 1 filter paper. A 20 mL aliquot of the filtrate was combined with 30 mL acetic acid and 1 mL saturated potassium iodide solution, and the mixture was kept in the dark for 30 min. Afterward, 1 mL of 1% starch solution and 50 mL distilled water were added. The solution was titrated against 0.005 N sodium thiosulfate until it became colorless. Peroxide value was calculated using the following equation (3):

(3) $$\mathrm{PV}\left(\frac{\mathrm{meq}}{\mathrm{kg}}\right)=\frac{V\times N\times 1000}{\text{Weight of sample}}$$

where *V* and *N* are the volume (mL) of 0.005 N sodium thiosulphate and the normality value of sodium thiosulphate, respectively.

#### 2.5.7 X‐Ray Diffraction (XRD)

The crystallinity of the cookie samples was analyzed using an X‐ray diffraction spectrometer (Bruker ADVANCE D8, Germany) equipped with CuKα1 radiation (λ = 1.540598 Å), following Walker et al. (2012). The operating conditions were: voltage 45 kV, current 40 mA, and room temperature. Diffraction patterns were collected in the 2θ range of 5°–80° with a step size of 0.1°.

#### 2.5.8 FTIR Spectroscopy

The FTIR spectra of cookie samples were obtained using an ATR‐FTIR spectrophotometer (Tensor II, Bruker) according to Alvarez‐Ramirez et al. (2020). Approximately 1 g of sample was placed on the crystal surface, and spectra were recorded in the range of 400–4000 cm^−1^ with a resolution of 4 cm^−1^ and an accumulation of 32 scans.

### 2.6 Statistical Analysis

All data were expressed as mean ± standard deviation of triplicate determinations. One‐way analysis of variance (ANOVA) was performed using SPSS software (version 26.0), and significant differences among means were identified at *p* < 0.05 using Duncan's multiple range test. Independent samples t‐tests were employed to compare differences between two groups. The Friedman test (K‐related samples) was applied to evaluate the significance of changes over time during storage.

## 3 Results and Discussion

### 3.1 Characteristics of Emulgels

#### 3.1.1 Solvent Holding Capacity (SHC)

**FIGURE 1:**
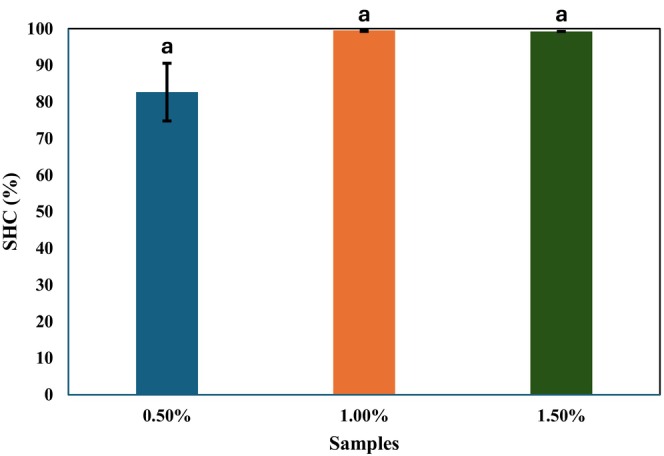
Solvent holding capacity (SHC) (%) of emulgels with different concentrations of κ‐carrageenan: 0.50%, 1.00%, and 1.50%. Different letters above the columns indicate a significant difference (*p* < 0.05).

SHC is an important indicator of gel network strength, particularly under mechanical stress. A high SHC value suggests structural integrity, while low SHC may result in oil leakage and subsequent degradation of the food matrix (Doan et al. 2018; Tavernier et al. 2018; Morales et al. 2023). The SHC of the emulgels ranged from 82.66% to 99.52%, with no significant differences among formulations (*p* > 0.05) (Figure 1). This indicates that SHC was not influenced by κ‐carrageenan concentration. The stabilization can be attributed to κ‐carrageenan interactions with hydrophobic regions of sunflower oil, which reduce interfacial tension and promote oil droplet stability within the aqueous phase (Salahi et al. 2023). Consequently, all emulgel formulations demonstrated sufficient SHC for potential application in food systems. These findings are consistent with previous reports. Yang et al. (2022) observed SHC values up to 93.13% in bigels containing κ‐carrageenan hydrogel and beeswax–corn oil oleogel. Similarly, Asyrul‐Izhar et al. (2023) reported SHC values above 91.80% in emulgels formulated with modified corn starch, canola oil, and gelatin.

#### 3.1.2 FTIR Spectroscopy

**FIGURE 2:**
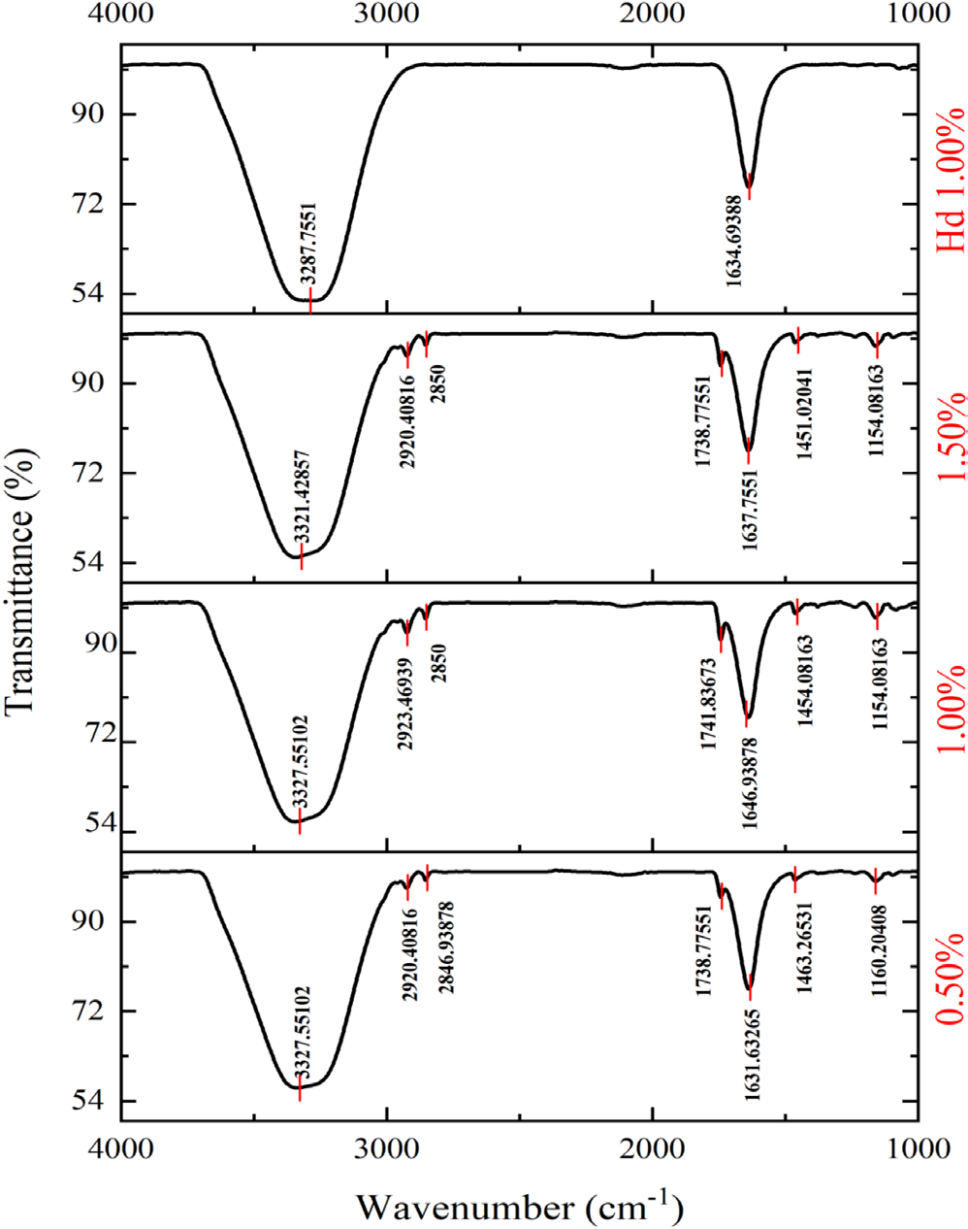
FTIR spectra of Hd 1.00%: Hydrogel phase containing 1.00% κ‐carrageenan, 1.50%: Emulgel containing 1.50% κ‐carrageenan, 1.00%: Emulgel containing 1.00% κ‐carrageenan, and 0.50%: Emulgel containing 0.50% κ‐carrageenan.

The FTIR spectra of the hydrogel phase (1.00% κ‐carrageenan) and emulgels (0.50%, 1.00%, and 1.50% κ‐carrageenan) were recorded in the range of 4000–400 cm^−1^ (Figure 2). All samples exhibited a broad peak at 3300 cm^−1^, corresponding to the stretching of O–H groups involved in hydrogen bonding (De Los Santos‐Trinidad et al. 2023). In the hydrogel, the peak appeared at 3287 cm^−1^ but shifted to a higher wavenumber (3320 cm^−1^) in the emulgels, indicating strong interactions between the hydroxyl groups of sunflower oil and κ‐carrageenan through hydrogen bonding, ultimately contributing to the stability of the emulgel. Small peaks were observed around 2920 and 2850 cm^−1^ in the emulgels, attributed to the C–H stretching vibrations of monoglycerides and triglycerides present in sunflower oil (Jeong et al. 2023; Ogbu and Ajiwe 2016). Additional peaks at 1740 and 1160 cm^−1^ were also identified in the emulgels, representing the C = O and C–O stretching of ester groups in triglycerides (Cakmak‐Arslan 2022). The ester stretching vibrations of κ‐carrageenan appeared at 1640 cm^−1^ across all samples. In the emulgel spectra, small peaks at 1450 cm^−1^ were detected, associated with C–H bending of CH_3_ groups and characteristic of sunflower oil (Adili et al. 2020). Overall, the emulgel samples displayed the characteristic peaks of both pure hydrogel and sunflower oil, confirming the coexistence of these components within the emulgels. Finally, broad peaks at 563 cm^−1^ (hydrogel) and 590 cm^−1^ (emulgels) were recorded, likely corresponding to vibrations of the backbone mode of α‐1,4‐glycosidic linkages. These features suggest effective interactions within the κ‐carrageenan matrix, which are essential for the structural stability and functional properties of the gel network (Lutsyk et al. 2024; Pozo et al. 2018).

#### 3.1.3 Mechanical Properties

The mechanical properties of the emulgels were evaluated in terms of hardness, adhesiveness, cohesiveness, springiness, gumminess, and chewiness at κ‐carrageenan concentrations of 1.00% and 1.50% (Table 2). The 0.50% κ‐carrageenan emulgel was excluded from this analysis because it lacked sufficient gel strength.

**TABLE 2:** Mechanical properties of 1.00% and 1.50% κ‐carrageenan emulgels.

Emulgel samples	Hardness (N)	Adhesiveness (mJ)	Cohesiveness	Springiness (mm)	Gumminess (g)	Chewiness (mJ)
1.00%	1.44 ± 0.00^b^	0.45 ± 0.05^a^	0.79 ± 0.00^a^	3.14 ± 0.00^a^	119.35 ± 0.91^b^	3.84 ± 0.24^a^
1.50%	3.25 ± 0.02^a^	1.57 ± 1.48^a^	0.53 ± 0.26^a^	2.76 ± 0.00^b^	174.55 ± 8.45^a^	4.73 ± 2.41^a^

*Note:* Mean ± standard deviation. Different letters in the same column show a significant difference between the samples (*p* < 0.05).

The 1.50% κ‐carrageenan emulgel exhibited significantly higher hardness (3.25 N) than the 1.00% emulgel (1.44 N) (*p* < 0.05), indicating that increasing κ‐carrageenan concentration leads to greater gel strength. This effect can be attributed to the availability of more κ‐carrageenan molecules capable of forming double‐helix structures as concentration increases. Similar findings were reported by Zampouni et al. (2023), who observed a significant increase in hydrogel hardness with rising κ‐carrageenan concentration.

Adhesiveness, defined as the resistance of the gel to detachment from a surface (Martins et al. 2019), was measured as 0.45 mJ for the 1.00% emulgel and 1.57 mJ for the 1.50% emulgel. However, this difference was not statistically significant (*p* > 0.05). Beyond κ‐carrageenan content, factors such as oil concentration and surface thickness also influence adhesiveness, and their combined effect likely contributed to the absence of significant differences between the two formulations.

Cohesiveness, which reflects the rigidity of internal bonds within the gel matrix (Nephomnyshy et al. 2020), was 0.79 for the 1.00% emulgel and 0.53 for the 1.50% emulgel. This difference was also not statistically significant (*p* > 0.05). The lack of significance may be explained by cohesiveness reaching a plateau at moderate κ‐carrageenan concentrations, due to network saturation effects and the molecular mechanisms of gel formation, including double‐helix structures and ionic crosslinking (Li et al. 2024).

Springiness decreased significantly with increasing κ‐carrageenan concentration (*p* < 0.05), measuring 3.14 mm and 2.76 mm for the 1.00% and 1.50% emulgels, respectively, showing an inverse relationship to hardness. The 1.00% emulgel displayed higher elasticity but lower strength, whereas the 1.50% emulgel formed a more brittle network that was more easily deformed. This trend aligns with observations by Zhang et al. (2021), who reported that increasing zein/carboxymethyl dextrin concentration in κ‐carrageenan‐based emulsion gels significantly enhanced hardness but reduced springiness.

Gumminess, which represents the perceived mouth sensation of semi‐solid materials and is influenced by hardness and cohesiveness (Chandra and Shamasundar 2015), was 119.35 g for the 1.00% emulgel and 174.55 g for the 1.50% emulgel, showing a significant difference (*p* < 0.05). Higher κ‐carrageenan concentration drives more helix formation and interchain association, producing a denser, stiffer gel network with higher hardness—therefore higher gumminess.

Chewiness, defined as the energy required to chew a semi‐solid material (Huang et al. 2007), also showed no significant difference between the 1.00% (3.84 mJ) and 1.50% emulgels (4.73 mJ) (*p* > 0.05). Nevertheless, Zampouni et al. (2023) reported that chewiness values increased significantly (*p* < 0.05) when κ‐carrageenan and gelatin concentrations were increased in hydrogels.

#### 3.1.4 Viscosity

**FIGURE 3:**
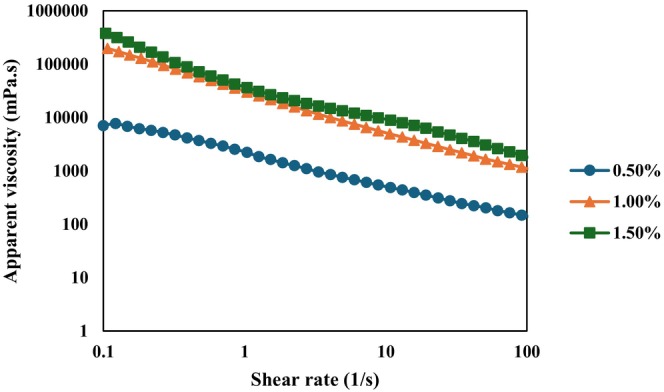
Apparent viscosity of 0.50% emulgel containing 0.50% κ‐carrageenan, 1.00% emulgel containing 1.00% κ‐carrageenan, and 1.50% emulgel containing 1.50% κ‐carrageenan.

The apparent viscosity of all emulgels decreased with increasing shear rate, demonstrating clear shear‐thinning behavior (Figure 3). This indicates that all samples behaved as pseudoplastic fluids, with their internal structures progressively disrupted during shearing (Chen et al. 2023). Similar findings were reported by Zhang et al. (2021), who observed shear‐thinning behavior in κ‐carrageenan‐based Pickering emulgels. The shear‐sensitive nature of emulsion gels is advantageous for food applications, as it enhances their spreadability (Patel et al. 2015). At low shear rates, the higher apparent viscosity of the emulgels can be attributed to extensive hydrogen bonding, which contributes to long‐term stability by reducing the likelihood of phase separation (Behera et al. 2015). Even at the highest shear rate (100 s^−1^), the apparent viscosity of the weakest emulgel remained significantly higher than that of liquid edible oils (Yalcin et al. 2012).

The viscosity of the emulgels increased consistently with rising κ‐carrageenan concentration. Specifically, the 0.50% κ‐carrageenan emulgel exhibited the lowest apparent viscosity. As a biopolymer, κ‐carrageenan has a pronounced thickening effect; thus, increasing its concentration predictably enhances viscosity by promoting a denser gel network that interacts strongly with sunflower oil. This interaction results in tighter binding between the hydrogel phase and oil droplets, ultimately improving the structural stability of the emulgels (Chen et al. 2020; Huang et al. 2024).

#### 3.1.5 Dynamic Rheological Measurements

**FIGURE 4:**
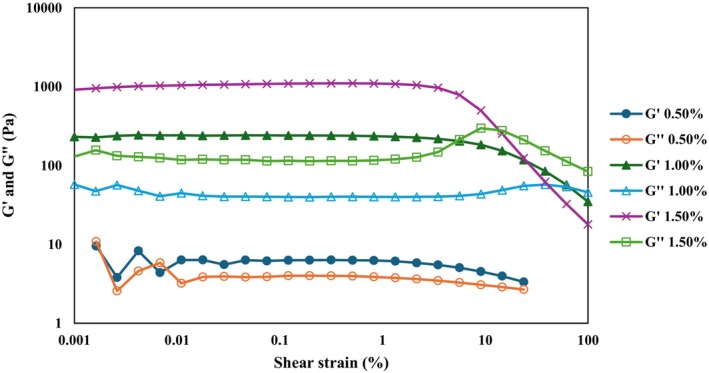
Amplitude sweeps of 0.50%: Emulgel containing 0.50% κ‐carrageenan, 1.00%: Emulgel containing 1.00% κ‐carrageenan, and 1.50%: Emulgel containing 1.50% κ‐carrageenan.

Figure 4 presents the amplitude sweeps of emulgels prepared with different κ‐carrageenan concentrations (0.50%, 1.00%, and 1.50%). The storage modulus (G′) represents the material's ability to store energy, reflecting its solid‐like character, whereas the loss modulus (G″) reflects energy dissipation, corresponding to viscous, fluid‐like behavior (dos Santos Carvalho et al. 2023).

An increase in κ‐carrageenan concentration from 0.50% to 1.50% led to a marked enhancement of the elastic response, indicating that higher κ‐carrageenan levels impart greater elasticity to the emulgels. For the 1.00% and 1.50% κ‐carrageenan emulgels, G′ values were approximately 10^2^ and 10^3^ Pa, respectively, and were significantly higher than their corresponding G″ values. This indicates that these systems exhibited predominantly solid‐like rather than fluid‐like behavior. In contrast, the 0.50% κ‐carrageenan emulgel displayed G′ values below 10 Pa, which were not significantly higher than G″, suggesting a weak viscoelastic structure. Moreover, the 0.50% κ‐carrageenan emulgel exhibited distinct crossover points (G′ = G″) at very low shear strain (< 0.01%), reflecting structural deformability even under minimal stress. By comparison, the 1.50% κ‐carrageenan emulgel formed a denser and more rigid gel network, characterized by a reduced linear viscoelastic region and greater brittleness. This was evident in its earlier crossover point at lower shear strain (14.6%), compared with 61.8% for the 1.00% κ‐carrageenan emulgel (Geonzon et al. 2023).

**FIGURE 5:**
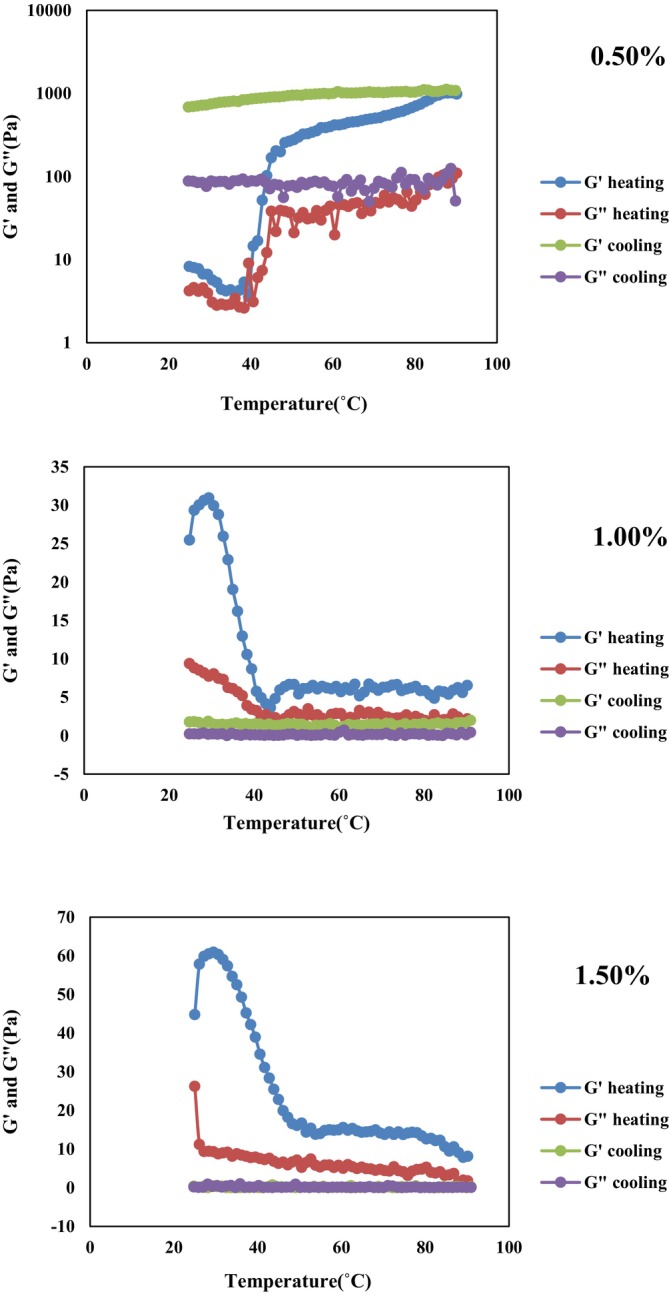
Changes in elastic (G') and viscous (G") moduli during heating and cooling, samples: 0.50%: Emulgel containing 0.50% κ‐carrageenan, 1.00%: Emulgel containing 1.00% κ‐carrageenan, and 1.50%: Emulgel containing 1.50% κ‐carrageenan.

During baking, changes in the rheological properties of emulgels, which are influenced by temperature, may strongly affect the physical characteristics and texture of the final product (Kim and Oh 2022). As shown in Figure 5, during heating, the moduli of all emulgels decreased sharply between 25°C and 40°C, corresponding to the melting of κ‐carrageenan. A similar behavior was observed in κ‐carrageenan‐based double emulsion gels, where G′ decreased sharply within the 10°C–40°C range during heating (Lee et al. 2023).

During heating, the 0.50% κ‐carrageenan emulgel initially exhibited G′ and G″ values of 8.2 and 4.2 Pa, respectively, with G′ > G″, indicating a weak gel‐like structure. A crossover was observed at approximately 42°C, likely reflecting rupture of the gel network; however, even beyond this point, G′ remained higher than G″. Liu et al. (2016) reported a similar crossover at 50.1°C in a 2% κ‐carrageenan hydrogel, which was followed by a gel–sol transition. In contrast, in the 0.50% emulgel, both moduli sharply increased after the crossover, possibly due to water loss from the gel matrix, leading to additional structural rearrangements (de Vries et al. 2018). This increase was not observed in the 1.00% and 1.50% emulgels, likely due to their higher κ‐carrageenan concentrations and greater ability to retain water. For the 1.00% κ‐carrageenan emulgel, G′ and G″ started at 25.4 and 9.3 Pa, respectively, and decreased gradually to 6.5 and 1.9 Pa at 90°C. Similarly, in the 1.50% emulgel, initial values of G′ and G″ were 44.7 and 26.2 Pa, respectively, decreasing to 8.13 and 1.77 Pa at 90°C. For both the 1.00% and 1.50% formulations, G′ and G″ remained nearly constant between 40°C and 90°C, and no crossover points were observed during heating.

During cooling, the 0.50% κ‐carrageenan emulgel maintained nearly constant moduli (with G′ > G″), and its elasticity was higher than at the start of heating, most likely due to water evaporation during heating. The 1.50% κ‐carrageenan emulgel, however, displayed multiple crossover points during cooling, reflecting its inability to reform a stable solid‐like network after disruption. This behavior suggests that high κ‐carrageenan concentrations create entangled networks that, once broken by heating, cannot reorganize into stable structures. The 1.00% emulgel showed no crossover points during cooling but exhibited reduced viscoelastic moduli, indicating partial recovery of the gel network (Liu et al. 2016).

Since no gel–sol transition was observed in the 1.00% κ‐carrageenan emulgel during either heating or cooling, this formulation may offer superior performance in baking applications, such as cookies, where structural stability during cooking and subsequent cooling is desirable.

#### 3.1.6 Microstructure Observation

**FIGURE 6:**
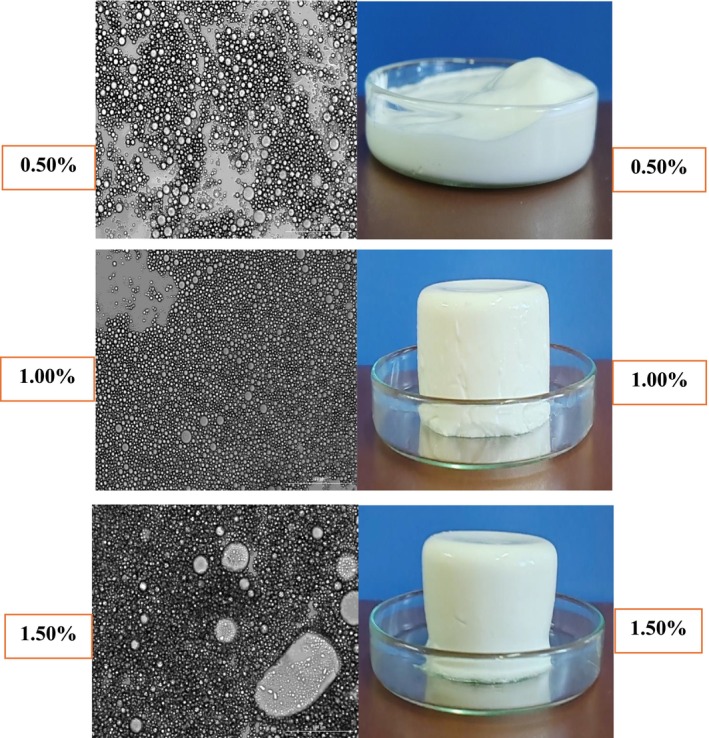
Microstructure and visual images of 0.50%: Emulgel containing 0.50% κ‐carrageenan, 1.00%: Emulgel containing 1.00% κ‐carrageenan, and 1.50%: Emulgel containing 1.50% κ‐carrageenan.

The microstructure and visual appearance of the emulgels are shown in Figure 6. Microscopy revealed spherical oil droplets in all emulgel samples, with droplet size decreasing as κ‐carrageenan concentration increased. This reduction in size can be attributed to the higher viscosity of the continuous phase, which restricts droplet coalescence. As a hydrocolloid gelling agent, κ‐carrageenan forms a stable and dense gel network within the dispersion medium (Lee et al. 2023). Thus, the addition of κ‐carrageenan enhances emulgel stability by providing both a thick protective coating around the oil droplets and a structured gel matrix in the continuous phase. These observations are consistent with the amplitude sweep results, which demonstrated that emulgels containing higher κ‐carrageenan concentrations (1.00% and 1.50%) exhibited greater gel strength than the 0.50% formulation. The visual images likewise confirmed the more structured appearance of the 1.00% and 1.50% κ‐carrageenan emulgels compared with the weaker 0.50% sample. Xu et al. (2020) reported a similar effect in emulsion gels, where higher concentrations of konjac glucomannan and inulin reduced oil droplet size in sodium caseinate–tea seed oil systems. In the 1.50% κ‐carrageenan emulgel, localized aggregation of oil droplets was observed, along with the presence of inner water droplets encapsulated within some oil droplets. Lee et al. (2023) reported comparable structures in κ‐carrageenan–methyl cellulose double emulsion gels, where internal water droplets were similarly retained within oil phases.

Because the 0.50% κ‐carrageenan emulgel lacked sufficient gel strength, only the 1.00% and 1.50% formulations were initially considered as shortening substitutes in cookie dough. However, the 1.50% emulgel could not be spread effectively due to its excessive hardness. This rigidity is likely due to the formation of more junction zones, producing a denser network resistant to deformation. Pehlivanoglu et al. (2017) also noted that hard gels can hinder dough processing and shaping in bakery applications, although in other products, such as frankfurters, the use of hard gels may be advantageous for improving structural strength (Ye et al. 2019). Consequently, the 1.00% κ‐carrageenan emulgel was selected as the fat replacer in cookie formulations. Shortening was substituted with this emulgel at two levels: 50% and 100%. The different cookie formulations were coded as follows: SH100—cookies made entirely with shortening; O100—cookies prepared with 100% liquid oil; SH50:E50—cookies containing equal proportions of shortening and emulgel; and E100—cookies in which all shortening was replaced with emulgel.

### 3.2 Characteristics of Cookies

#### 3.2.1 Fat Content

The fat content of control and fat‐reduced cookies is presented in Table 3. Incorporation of emulgel into the formulations resulted in a significant decrease in fat content after baking compared with the control cookies. In SH50:E50 cookies, the fat content decreased by 30% relative to SH100 (18.89% and 27.17%, respectively), while in E100 cookies the decrease was 50% relative to O100 (11.91% compared with 24.28%). According to European regulations (Union 2006), a minimum reduction of 30% in fat compared with conventional products qualifies the product as “reduced fat.” Therefore, both SH50:E50 and E100 cookies can be labeled as reduced‐fat products. This finding highlights the effectiveness of emulgel as a fat substitute in cookie formulations.

**TABLE 3:** Fat content and its variation in cookies during 3 months after baking.

Samples	After baking (g/100 g)	3 months after baking (g/100 g)	Diff (3 months after baking – after baking)
SH50: E50	18.89 ± 0.12^c^	17.56 ± 0.1^b^	−1.33 ± 0.007^c^
E100	11.91 ± 0.24^d^	11.07 ± 0.05^c^	−0.84 ± 0.2^b^
SH100	27.17 ± 0.12^a^	27.24 ± 0.09^a^	0.08 ± 0.02^a^
O100	24.28 ± 0.19^b^	6.03 ± 0.02^d^	−18.12 ± 0.17^d^

*Note:* Mean ± standard deviation. Different letters in the same column show a significant difference between the samples (*p* < 0.05). Samples: cookies formulated with 50% emulgel: 50% shortening (SH50: E50), 100% emulgel (E100), 100% shortening (SH100), and 100% oil (O100).

In contrast, the O100 cookies exhibited a pronounced fat loss during storage, with reductions exceeding 75% after three months (from 24.28% to 6.03%). This behavior can be attributed to the liquid nature of oil, which promotes migration and accumulation on the product surface. Such migration may adversely affect both texture and flavor over time. Conversely, the fat content of the reduced‐fat formulations remained relatively stable throughout storage. However, fat reduction in SH50:E50 cookies (1.33%) was more pronounced than in E100 (0.84%) (*p* < 0.05). This difference may stem from the partial breakdown of the emulgel structure during mixing with shortening in dough preparation.

Our results are consistent with the findings of Sharifi et al. (2025), who reported that cookies formulated with sunflower oil showed the greatest fat reduction compared to those made using shortening or κ‐carrageenan/monoglyceride bigels. Furthermore, in their study, the fat content of bigel‐based cookies remained nearly stable throughout three months of storage.

#### 3.2.2 Hardness Evaluation of Cookies

The hardness of the cookie samples during storage is presented in Table 4. One day after baking, the hardness of SH100 (25.36 N) and SH50:E50 (21.51 N) cookies was significantly higher than that of E100 (12.88 N) and O100 (14.12 N) (*p* < 0.05). Shortening contributes to harder cookies because, as a semi‐solid fat at room temperature, it can be uniformly dispersed in the dough, thereby providing a crisp and firm texture (Shi et al. 2025). By contrast, sunflower oil reduced cookie hardness, likely because dough prepared with liquid oil is smoother and less rigid than dough prepared with shortening (Sharifi et al. 2025). Similar findings were reported by Lim et al. (2023), who replaced shortening with emulgel based on pea protein isolate and soybean oil; in their study, increasing emulgel substitution reduced hardness, with the lowest values observed in cookies containing 100% emulgel immediately after production.

**TABLE 4:** Hardness (N) analysis of cookies during the time.

Samples	Day 1	Day 10	Day 20	Day 30	Diff (Day 30—Day 1)
SH50: E50	21.51 ± 0.39^aA^	21.56 ± 2.25^aA^	27.35 ± 0.87^aA^	18.38 ± 6.21^bA^	−3.14 ± 6.61^b^
E100	12.88 ± 1.98^bB^	19.45 ± 0.05^aAB^	32.79 ± 6.44^aA^	30.70 ± 5.80^aA^	17.83 ± 3.81^a^
SH100	25.36 ± 1.28^aA^	23.43 ± 0.20^aA^	18.44 ± 3.18^bAB^	18.72 ± 1.81^bB^	−6.64 ± 2.70^b^
O100	14.12 ± 4.44^bAB^	20.91 ± 0.33^aA^	10.68 ± 3.58^bB^	12.14 ± 0.90^bB^	−1.53 ± 5.97^b^

*Note:* Mean ± standard deviation. Different small letters in the same column and differenr capital letters in the same row, show significant differences between the samples (*p* < 0.05). Samples: cookies formulated with 50% emulgel: 50% shortening (SH50: E50), 100% emulgel (E100), 100% shortening (SH100), and 100% oil (O100).

During 30 days of storage, SH100 cookies showed a decrease in hardness (from 25.36 N to 18.72 N), whereas E100 cookies exhibited a significant increase (from 12.88 N to 30.70 N) (*p* < 0.05). This increase in hardness may be attributed to the moisture‐retention and stabilizing effects of the emulgel, which likely promoted stronger network formation within the cookie matrix, thereby enhancing structural firmness over time. On the other hand, the hardness of SH50:E50 cookies remained relatively stable, as the variations observed during storage were not statistically significant. This indicates that partial replacement of shortening with emulgel (50%) maintained the desirable texture properties more effectively than full replacement.

These findings suggest that the SH50:E50 formulation is the most suitable for shortening substitution in cookies, balancing texture stability with reduced fat content. In agreement, Sharifi et al. (2025) reported that cookies prepared with 50% κ‐carrageenan/monoglyceride bigel exhibited no significant hardness changes during 29 days of storage.

#### 3.2.3 Moisture Content of Cookies

The moisture content of the cookies is presented in Table 5. One day after baking, E100 exhibited the highest moisture content (10.07%), while the other formulations showed no significant differences. This higher moisture level in E100 can be attributed to the complete replacement of shortening with emulgel, which naturally contributes additional water to the formulation.

**TABLE 5:** Moisture content (%) of cookies during the time.

Samples	Day 1	Day 8	Day 15	Day 22	Day 29	Diff (Day 29—Day 1)
SH50: E50	5.26 ± 0.05^bA^	5.48 ± 0.23^cA^	5.04 ± 0.11^bcA^	5.24 ± 0.04^dA^	5.77 ± 0.39^bA^	0.51 ± 0.45^a^
E100	10.07 ± 0.01^aA^	11.27 ± 0.21^aA^	8.88 ± 0.24^aA^	8.80 ± 0.10^aA^	11.58 ± 2.66^aA^	1.50 ± 2.68^a^
SH100	4.46 ± 0.82^bA^	5.16 ± 0.05^cA^	4.65 ± 0.32^cA^	5.64 ± 0.02^cA^	6.00 ± 0.12^bA^	1.54 ± 0.94^a^
O100	6.16 ± 1.18^bA^	6.23 ± 0.09^bA^	5.33 ± 0.13^bA^	6.83 ± 0.25^bA^	7.23 ± 0.09^bA^	1.07 ± 1.28^a^

*Note:* Mean ± standard deviation. Different small letters in the same column and different capital letters in the same row, show significant differences between the samples (*p* < 0.05). Samples: cookies formulated with 50% emulgel: 50% shortening (SH50: E50), 100% emulgel (E100), 100% shortening (SH100), and 100% oil (O100).

Over the course of one month, the moisture content of all samples remained relatively stable. Also, overall moisture variations among all formulations were not statistically significant, suggesting that incorporating emulgel at either 50% or 100% substitution levels did not substantially affect changes in cookie moisture during storage.

These results are consistent with previous studies. Giarnetti et al. (2015) reported that cookies formulated with 50% and 100% of an emulsion‐filled gel based on inulin and extra virgin olive oil had significantly higher moisture contents (4.5 and 7.1 g/100 g, respectively) compared with conventional cookies (3.7 g/100 g).

#### 3.2.4 Color

**FIGURE 7:**
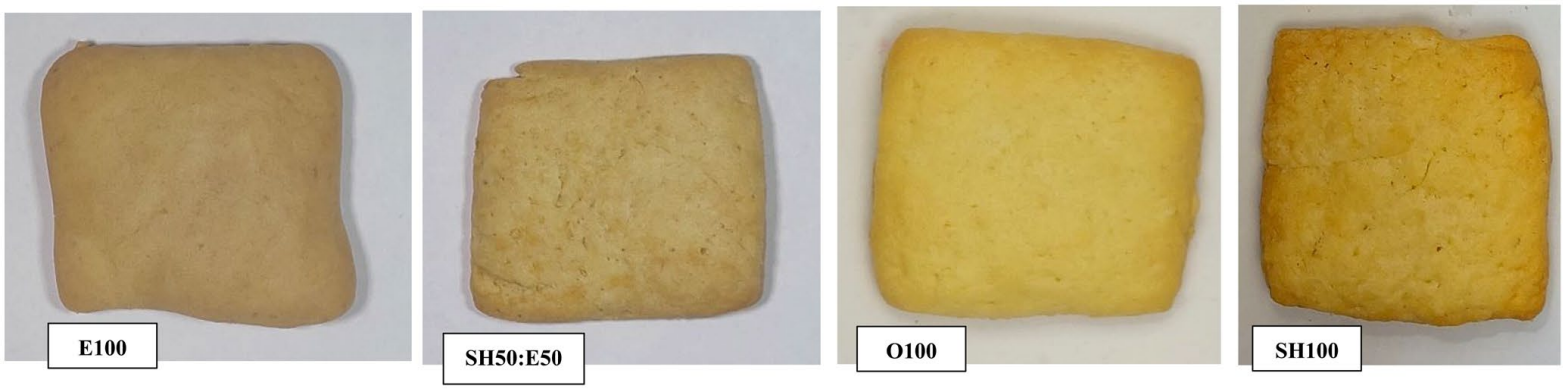
Photographs of cookie samples, samples: Cookies formulated with 100% emulgel (E100), 50% emulgel: 50% shortening (SH50: E50), 100% oil (O100), 100% shortening (SH100).

Photographs of the cookie samples are shown in Figure 7. Analysis of the color parameters provided insight into how different fat sources and emulgel substitution influenced cookie appearance. The corresponding L*, a*, and b* values are presented in Table 6, where L* denotes lightness (0 = black, 100 = white), a* represents the green (−90) to red (+90) axis, and b* represents the blue (−90) to yellow (+90) axis (Giuffrè et al. 2022).

**TABLE 6:** Cooking loss (%) and color parameters of cookies.

Samples	Cooking loss (%)	L*	a*	b*
SH50: E50	19.53 ± 1.30^a^	66.10 ± 1.91^c^	2.50 ± 0.84^c^	33.70 ± 1.41^c^
E100	19.85 ± 1.65^a^	60.50 ± 1.90^d^	5.80 ± 0.91^a^	29.80 ± 1.47^d^
SH100	16.13 ± 0.76^b^	72.30 ± 2.49^b^	4.50 ± 1.77^b^	44.70 ± 4.05^a^
O100	14.43 ± 0.81^c^	74.60 ± 2.50^a^	3.80 ± 1.22^b^	40.60 ± 1.89^b^

*Note:* Mean ± standard deviation. Different letters in the same column show a significant difference between the samples (*p* < 0.05). Color parameters: L*: lightness, a*: redness/greenness, b*: yellowness/blueness. Samples: cookies formulated with 50% emulgel: 50% shortening (SH50:E50), 100% emulgel (E100), 100% shortening (SH100), and 100% oil (O100).

The results showed that O100 had the highest L* value, while E100 exhibited the lowest (74.60 and 60.50, respectively) (*p* < 0.05). Lightness decreased progressively with increasing emulgel substitution, indicating that incorporation of emulgel produced darker cookies. In baked products, such darkening is commonly associated with Maillard reactions and caramelization.

For the a* parameter, E100 displayed the highest value, whereas SH50:E50 recorded the lowest (5.80 and 2.50, respectively) (*p* < 0.05). By contrast, SH100 and O100 did not differ significantly in a* (*p* > 0.05). Regarding b*, SH100 showed the highest yellowness value (44.70), followed by O100 (40.60), while E100 exhibited the lowest (29.80) (*p* < 0.05). This trend can be attributed to the presence of carotenoid pigments in shortening and sunflower oil, which contribute to yellow coloration. Thus, replacing these fats with emulgel reduces b* values and diminishes yellowness.

Barragán‐Martínez et al. (2022) reported that replacing shortening with a bigel composed of candelilla wax–canola oil (oleogel) and gelatinized corn starch (hydrogel) increased L* and b* values but decreased a*. Such variations highlight that the color outcomes are strongly dependent on the types of polysaccharide and fat sources employed in the formulation.

#### 3.2.5 Cooking Loss

The cooking loss values are presented in Table 6. The highest values were observed for E100 and SH50:E50, with no significant differences between these two formulations (19.85% and 19.53%, respectively). This indicates that incorporating emulgel at either 50% or 100% substitution did not significantly affect cooking loss. In contrast, O100 exhibited the lowest cooking loss (14.43%), which may be attributed to the reduced water content in its dough, resulting in minimal weight loss during baking (Quilaqueo et al. 2022).

#### 3.2.6 Peroxide Value

Peroxide values were recorded after approximately four months of storage (Table 7). On the 50th day, no significant differences were detected among all cookie samples. By day 90, however, SH100 exhibited the highest peroxide value, followed by O100 (1.92 meq/kg and 1.37 meq/kg, respectively) (*p* < 0.05). In contrast, SH50:E50 and E100 maintained the lowest and most stable peroxide values throughout storage. This stability can be attributed to the reduced fat content of these formulations compared to the controls. These findings highlight that emulgel incorporation can enhance the oxidative stability of unsaturated oils in bakery products, which otherwise poses a challenge for the direct use of edible vegetable oils (Tanislav et al. 2023).

**TABLE 7:** Peroxide value (meq/kg) of cookies during 4 months of storage.

Samples	Day 50	Day 70	Day 90	Day 110
SH50: E50	0.60 ± 0.26 ^aA^	0.47 ± 0.08 ^bA^	0.66 ± 0.11 ^cA^	0.45 ± 0.06 ^abA^
E100	0.45 ± 0.06 ^aA^	0.50 ± 0.00 ^bA^	0.58 ± 0.06 ^cA^	0.56 ± 0.09 ^aA^
SH100	0.49 ± 0.07 ^aB^	0.54 ± 0.07 ^bB^	1.92 ± 0.10 ^aA^	0.37 ± 0.05 ^bB^
O100	0.69 ± 0.14 ^aBC^	0.89 ± 0.12 ^aB^	1.37 ± 0.17 ^bA^	0.41 ± 0.00 ^abC^

*Note:* Mean ± standard deviation. Different small letters in the same column and different capital letters in the same row show significant differences between the samples (*p* < 0.05). Samples: cookies formulated with 50% emulgel: 50% shortening (SH50:E50), 100% emulgel (E100), 100% shortening (SH100), and 100% oil (O100).

After day 90, the peroxide values of all samples decreased, likely due to the degradation of primary peroxide compounds into secondary oxidation products. For this reason, measurements were not extended beyond this point.

#### 3.2.7 XRD

**FIGURE 8:**
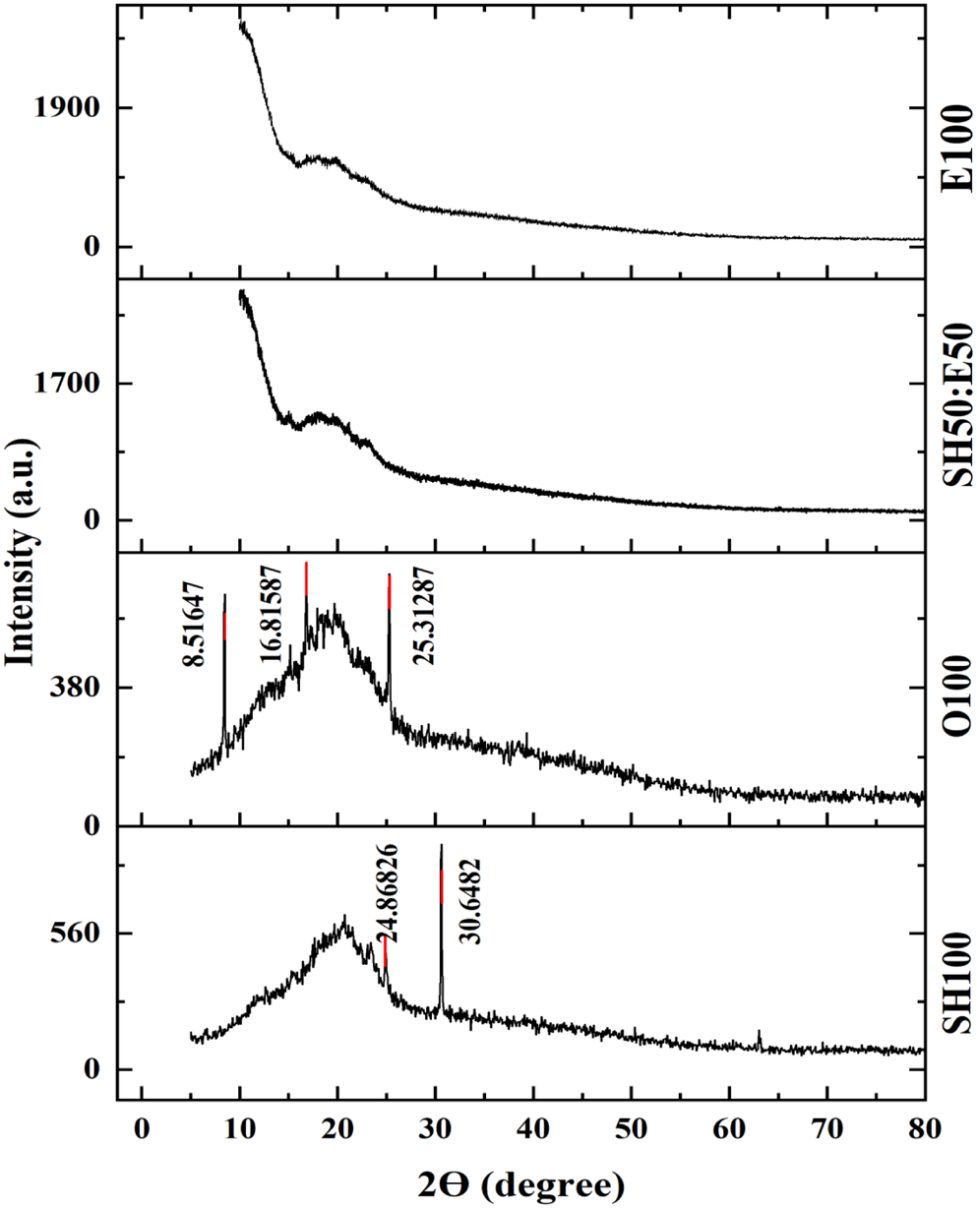
XRD pattern of cookie samples, samples: Cookies formulated with 100% emulgel (E100), 50% emulgel: 50% shortening (SH50: E50), 100% oil (O100), 100% shortening (SH100).

The XRD diffractograms are presented in Figure 8. Distinct diffraction peaks were observed at 8.51°, 16.81°, and 25.31° for O100, and at 24.86° and 30.64° for SH100. These patterns indicate that both O100 and SH100 exhibited higher crystallinity compared to the emulgel‐based formulations, which showed broader, hump‐like peaks. Relative crystallinity values further supported this observation: SH50:E50 and E100 displayed lower crystallinity (13.71% and 15.15%, respectively), whereas O100 and SH100 showed higher crystallinity (28.16% and 25.71%, respectively).

These results suggest that the incorporation of emulgels reduces lipid–amylose complex formation, likely due to their lower fat content. Chen et al. (2024) reported that increased crystallinity is associated with reduced digestible starch content in cookies. Therefore, substituting shortening with emulgels may improve digestibility properties compared to conventional cookies.

#### 3.2.8 FTIR

**FIGURE 9:**
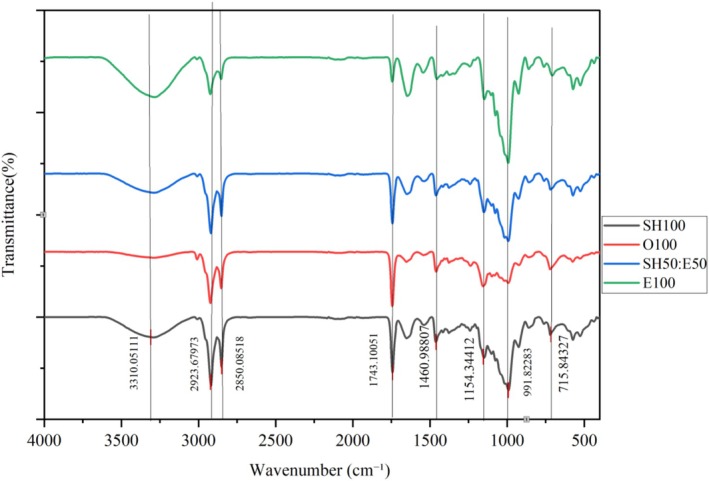
FTIR pattern of cookie samples, samples: Cookies formulated with 100% shortening (SH100), 100% oil (O100), 50% emulgel: 50% shortening (SH50:E50), 100% emulgel (E100).

The FTIR spectra of cookie samples are displayed in Figure 9. Major peaks were identified at 3310, 2923, 2850, 1743, 1460, 1154, 991, and 715 cm^−1^. The broad peak at 3310 cm^−1^ corresponds to hydroxyl groups, indicating hydration in the cookies. Among the samples, E100 exhibited the most pronounced band at this wavenumber, suggesting stronger hydrogen bonding due to the presence of κ‐carrageenan, which can interact with water and form gels. In contrast, O100 showed the weakest intensity at 3310 cm^−1^, reflecting fewer hydrogen bonds and functional groups. The region between 3000 and 2500 cm^−1^ is associated with C–H stretching, where peaks at 2923 and 2850 cm^−1^ represent C–H bonds in triglyceride molecules (Tyagi and Chauhan 2020). As fat content decreased, the intensity of these peaks diminished, particularly in the E100 sample. Peaks at 1743 and 1460 cm^−1^ correspond to C = O stretching in amides and N–H stretching of methyl groups (Pradhan et al. 2023). Vibrations in the 1111–1197 cm^−1^ range are attributed to C–O, C–O–C, and C–C stretching of glycosidic bonds. Additionally, the band at 991 cm^−1^ was associated with C–OH bending vibrations, which are moisture‐sensitive (Zhang et al. 2024). This peak appeared with greater intensity in E100, consistent with its higher moisture content. Finally, bands between 800 and 600 cm^−1^ were assigned to C = C bending vibrations in the aromatic ring of glucose pyranose (Gani et al. 2021).

Importantly, while peak intensities varied among formulations, all FTIR spectra exhibited bands at the same positions, indicating that the incorporation of emulgels did not alter the fundamental cookie matrix structure.

## 4 Conclusion

This study investigated the properties of κ‐carrageenan‐based emulgels and their application as fat replacers in cookies. The results demonstrated that altering the κ‐carrageenan concentration did not affect solvent‐holding capacity. However, texture analysis revealed that increasing the κ‐carrageenan concentration led to greater hardness, whereas springiness showed an opposite trend. Apparent viscosity also increased with higher κ‐carrageenan levels. Rheological analysis confirmed that greater κ‐carrageenan content enhanced the elastic behavior of emulgels, indicating increased structural resilience at higher concentrations. During heating and cooling, the 1.00% κ‐carrageenan emulgel exhibited no gel–sol transition but showed reduced viscoelastic parameters, reflecting partial network recovery. In contrast, the 0.50% and 1.50% formulations displayed multiple crossover points during thermal cycling. Furthermore, higher κ‐carrageenan concentrations were associated with smaller oil droplet sizes in the emulgels.

Based on these findings, the 1.00% κ‐carrageenan emulgel was selected as a fat replacer in cookie formulations at 50% (SH50:E50) and 100% (E100) substitution levels. Fat analysis revealed that SH50:E50 cookies showed a 30% reduction in fat content compared to SH100, while E100 cookies demonstrated a 50% reduction relative to O100. Over one month of storage, SH50:E50 cookies maintained hardness levels comparable to control formulations with shortening or oil, whereas E100 cookies exhibited a significant increase in hardness. Moisture content remained largely stable across all formulations, and emulgel incorporation did not significantly alter moisture retention during storage. Regarding color, L* values decreased with increasing emulgel substitution, and the highest b* values were recorded in control cookies, reflecting the presence of carotenoid pigments in shortening and sunflower oil. XRD analysis indicated that control cookies had higher relative crystallinity than emulgel‐based cookies, suggesting reduced lipid–amylose complex formation in the latter.

Considering both physical and textural attributes, the SH50:E50 formulation was identified as the most suitable approach, as it maintained stable hardness during storage while achieving substantial fat reduction. Although this study did not directly assess health‐promoting properties, reducing fat content in cookies may contribute to improved nutritional profiles. Importantly, this is the first study to report the use of κ‐carrageenan–sunflower oil emulgels as fat replacers in cookies. Future research should incorporate sensory evaluation, extend application to broader bakery systems, and investigate metabolic mechanisms and health impacts, thereby advancing the development of low‐fat, functional baked products.

## Author Contributions

**Mahdieh Sharifi:** conceptualization (equal), formal analysis (equal), investigation (equal), methodology (equal), software (equal), writing – original draft (equal). **Niloofar Moshfegh:** conceptualization (equal), investigation (equal), methodology (equal), writing – original draft (equal). **Azam Abbasi:** conceptualization (equal), funding acquisition (equal), investigation (equal), methodology (equal), supervision (equal), validation (equal), writing – review and editing (equal).

## Ethics Statement

The protocol of the study was approved by the ethics committee at Shiraz University of Medical Sciences and was registered at IRCT with ID number IR.SUMS.SCHEANUT.REC.1403.061.

## Conflicts of Interest

The authors declare no conflicts of interest.

## Data Availability

Data will be made available on request.
